# Supplementary material for: The causal relationship between the human gut microbiota and pyogenic arthritis: a Mendelian randomization study
Source: Front Cell Infect Microbiol. 2024 Nov 26;14:1452480. doi: 10.3389/fcimb.2024.1452480 (PMC11629706; doi:10.3389/fcimb.2024.1452480)
Supplement: Supplementary file 1 [file DataSheet1.zip › mendelian test/ebi-a-GCST90027773.csv_funnel.pdf]

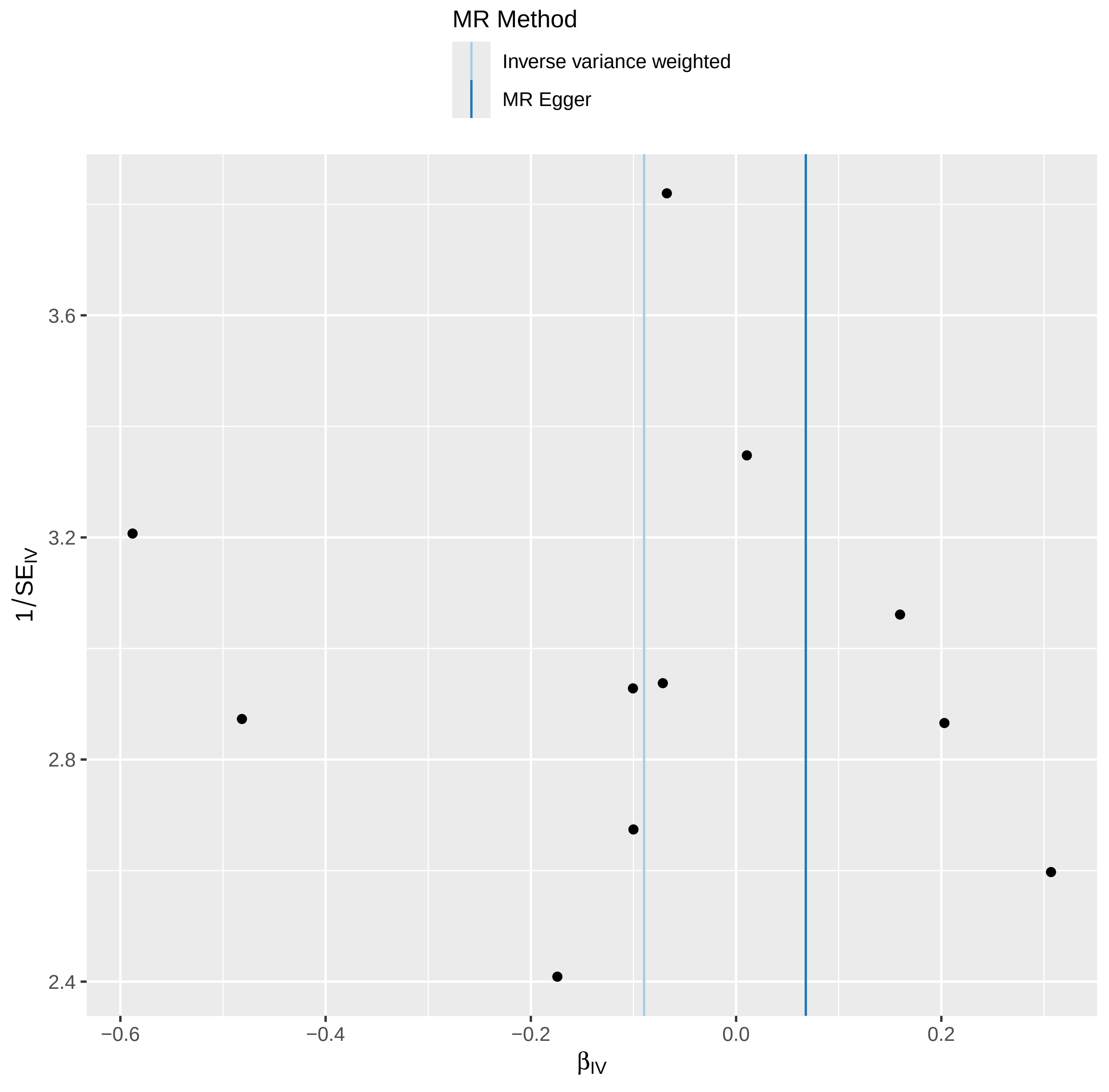
MR Method
Inverse variance weighted
MR Egger
3.6
3.2
2.8
2.4
1/SE_IV
−0.6
−0.4
−0.2
0.0
0.2
β_IV
